# Does personality moderate the association between social involvement and personal recovery in psychosis?

**DOI:** 10.1186/s12888-024-06372-0

**Published:** 2024-12-27

**Authors:** Pien Leendertse, David van den Berg, Stynke Castelein, Cornelis Lambert Mulder

**Affiliations:** 1 Youz, Institute for Mental Healthcare, Parnassia Psychiatric Institute, Rotterdam, The Netherlands; 2 Department of Psychiatry, Erasmus University Medical Center, Rotterdam, The Netherlands; 3 Department of Clinical Psychology, VU University and Amsterdam Public Health Research Institute, Amsterdam, The Netherlands; 4 Research and Innovation, Parnassia Psychiatric Institute, The Hague, The Netherlands; 5 Lentis Research, Lentis Psychiatric Institute, Groningen, The Netherlands; 6 Faculty of Behavioral and Social Sciences, Clinical Psychology and Experimental Psychopathology, University of Groningen, Groningen, The Netherlands; 7 Antes, Institute for Mental Healthcare, Parnassia Psychiatric Institute, Rotterdam, The Netherlands; 8 GGZ Westelijk Noord-Brabant, Institute for Mental Healthcare, Postbus 371, Bergen op Zoom, 4600 AJ The Netherlands

**Keywords:** Personal recovery, Social involvement, Psychosis, Personality traits

## Abstract

**Objective:**

Social factors are central in personal recovery (PR) and treatment of psychosis. However, weak associations between social involvement and PR were found. We aimed to replicate this weak association, and test whether it was explained by a moderating effect of neuroticism and extraversion.

**Method:**

This cross-sectional study included 284 psychotic disorder patients. PR was assessed using the Recovery Quality of Life (ReQoL) questionnaire. Social involvement with a formative measure of the frequency of social interaction, and neuroticism and extraversion with the NEO Five Factor Inventory (NEO-FFI).

**Results:**

A small direct effect of social involvement on PR (β=−0.24, *p* < 0.001) was found, explaining 6% of the variance in PR. The addition of neuroticism (β=−0.60, *p* < 0.001) predicted 41% of variance in PR; extraversion (β = 0.34, *p* < 0.001) predicted 16%. We did not observe a moderating effect of neither neuroticism (β=-0.06, *p* = 0.232), nor extraversion (β = 0.01, *p* = 0.956).

**Conclusion:**

The weak association between social involvement and PR could not be explained by the moderating effect of neuroticism or extraversion. The increase in explained variance in PR implies that neuroticism is associated with PR in a direct and clinically relevant way. This emphasizes the importance of attending to negative emotions and underlying stressors in treatment of psychosis.

## Introduction

Recovery is a broad concept, consisting of different, but interrelated processes, e.g. the reduction of symptoms, improvement in cognitive functioning, the improvement in psychosocial functioning and living a meaningful life. Thus, recovery from psychosis entails more than the reduction of psychotic symptoms. When living with a psychotic disorder, people can feel recovered while still experiencing psychotic symptoms (e.g. when hallucinations are perceived in a less stressful way) and vice versa; conversely, the absence of symptoms does not necessarily mean people feel recovered [3]. This subjective experience of recovery, which is often referred to as personal recovery (PR), is described in the widely endorsed conceptual framework CHIME, an acronym representing five complementary processes that, according to people with severe mental illness (SMI), are instrumental to PR: (1) Connectedness; (2) Hope and optimism about the future; (3) Identity and overcoming stigma; (4) Meaning in life; and (5) Empowerment [16]. Symptoms, adverse childhood experiences and other environmental stressors might have a negative impact on these processes. Moreover, different social aspects of people’s experience are important to several of the elements of PR, particularly Connectedness, which includes interpersonal relationships and social inclusion; and Identity, whose rebuilding mostly takes place within the context of stigma and discrimination [26].

Of all patients with SMI, those with psychosis battle with the highest levels of public stigma. Furthermore, they experience high rates of trauma, and, in many cases, limited social networks [14, 39]. It was shown by a thematic synthesis of qualitative research from the patients’ perspective [37] that these patients face two particular barriers to PR: social exclusion and stigma, whether perceived or internalized.

The role of social factors in the onset of psychosis has been well established. The factors in question include social exclusion [25], childhood adverse events [34], and social rank [37]. As a result, an increasing body of recovery-oriented interventions in psychosis now focus on interpersonal factors and social functioning. Some examples are family interventions, including psychoeducation about psychosis and engaging both patients and family in this [7], resource groups [27] and individual placement and support [10].

Although such interventions proceed from the principle that recovery is promoted by strengthening social networks and social functioning, a meta-analysis of factors associated specifically with PR in psychosis found relatively weak effect sizes for its relationship with social factors (social support 0.28; work & housing 0.23; and psychosocial functioning 0.31) [18]. These weak associations might indicate that social involvement either has a limited effect on PR, or that it may affect PR differently in different people. One such difference among psychotic disorder patients may be their personality traits.

Although we could not find studies on the associations between personality traits and PR in psychotic disorder patients, some studies investigated the associations between personality traits and Quality of Life (QoL) in both the general population [22] and in psychotic disorder patients. QoL is sometimes viewed as an aspect of PR. A systematic review of the influence of personality traits on QoL in psychotic disorder patients concluded that neuroticism and extraversion were the traits associated with QoL [13]. More specifically, impaired QoL was found to be associated with high levels of neuroticism in people with psychosis [4, 5, 24]. This was confirmed by Van Dijk et al. [31], who found that higher levels of neuroticism and lower levels of extraversion were associated with lower QoL. Of note, both associations remained stable over time.

Next to the wider literature on the association between personality traits and social functioning [21], mixed results were found regarding this in people with psychosis. Social functioning is a broad concept, which is based on both an objective, quantifiable dimension (e.g., such as the ability to meet the social roles associated with being a family member or friend, or vocational engagement); and a subjective dimension comprising self-reported measures of social roles and measures of satisfaction (or of the problems experienced) with family life, recreational activities, and life as a whole [23]. To avoid conceptual overlaps with the subjective measurement of PR (such as Connectedness in CHIME), and with the subjective rating of social interaction in personality traits, we limited social functioning to objective, quantifiable dimensions of social involvement. While the study of Boyette et al. [6] indicates a strong role for extraversion in social involvement (e.g. withdrawal), Lysaker and Davis [19] found no association at all between extraversion and social involvement (e.g. the frequency of social contact). 

Taken together, neuroticism and extraversion may thus be the important factors to focus on when studying the associations between social involvement and PR in psychosis. In this study, we therefore aim to provide insight into the association between social involvement and PR, exploring how it may be influenced by neuroticism and extraversion. Since extraversion indicates a higher degree of sociability, positive emotionality and general activity, we hypothesize it to have a positive moderating effect on the association between social involvement and PR. While neuroticism, indicating the susceptibility to negative affect and psychological distress, and therefore potentially the tendency to withdraw from social interaction, to have a negative moderating effect.

Therefore, we expected: (1) the association between social involvement and PR to be relatively weak, with neuroticism and extraversion having opposite moderating effects on the association, namely; (2) that extraversion would strengthen the association between social involvement and PR; while we expected (3) neuroticism would weaken the association between social involvement and PR.

## Methods

### Participants and study design

This cross-sectional exploratory study used baseline data from the UP’S study [30], and was reported according to the STROBE guidelines [36]. UP’S is an observational cohort study intended to investigate recovery processes in people with a non-affective psychotic disorder. Participants are followed for ten years. Every year, an assessment takes place in which interactions between different dimensions of recovery are explored (clinical, functional, social and personal recovery). Furthermore, potential determinants of the different recovery dimensions are explored, including for example personality traits. In the current study, we selected the baseline data of participants in UP’S recruited between October 2017 and December 2022.

Participants were recruited mainly from outpatient teams of ten mental healthcare institutions in the southwestern Netherlands. The teams that participated in the UP’S study consisted of specialized early-episode psychosis services, and Flexible Assertive Outreach Teams (FACT) that provided care for SMI patients, including psychotic disorder patients. The UP’S study included patients aged between 18 and 65 years, who had a primary diagnosis of a schizophrenia-spectrum disorder according to DSM-5. Patients with insufficient proficiency of the Dutch language were excluded.

To ensure that inclusion in the study was independent of symptom severity, eligible participants who fulfilled the inclusion criteria at each participating location were selected randomly through a search in the electronic patient files. After evaluation by the clinician involved, student researchers (master’s students in psychology or medicine) approached patients to provide information on the study, and to ask their informed consent to participate. These student researchers participated in the teams, and were trained and supervised with regard to assessing, scoring and entering data. See Van Aken et al. [30] for an extensive description of the study protocol. 

The UP’S study was approved by the Regional Committee for Medical and Health Research Ethics in Rotterdam (Registration code: MEC-2017-340).

### Measures

#### Sociodemographic and patient characteristics

Sociodemographic data comprised age, gender, employment status, living situation (e.g., alone, with partner, or in an mental health care (MHC) institution). The following patient characteristics were collected: DSM-5 diagnosis, Positive and Negative Symptom Scale-Remission Tool (PANSS-R, [1]) scores, and duration of care (years since first contact with MHC).

#### Personal recovery

The outcome measure used to examine PR was the Recovery Quality of Life 10-item version (ReQoL-10), a user-friendly patient-reported outcome measure [15]. This questionnaire contains themes related to concepts of quality of life and recovery in accordance with the CHIME framework, such as hope, belonging and relationships, and autonomy [16]; although it was not validated against the CHIME framework, it was recommended as one of the patient-reported outcome measures for PR by the Psychotic Disorders Working Group of the International Consortium for Health Outcomes Measurement [20]. Items score from 0 (none of the time) to 4 (most of the time), or the reverse if items are worded negatively. Sum scores range from 0 (low degree of recovery) to 40 (high degree of recovery). If at least nine out of ten scores had been recorded, missing values were imputed on the basis of the participant’s mean score.

For this study we used the Dutch translation of the ReQoL, which showed good internal consistency estimates (Cronbach’s alpha = 0.862, McDonald’s Omega = 0.863) [29].

#### Social involvement

In this study, we conceptualized social involvement as the amount of interaction with others in society, as an aspect of social functioning. Like social functioning, interaction with others has an objective dimension (the frequency of interactions) and a subjective dimension (the perceived quality of these interactions). To quantify social involvement, we selected three questions within the UP’S study, that identified the frequency of interactions with others: (1) “How often do you meet friends or good acquaintances?”; (2) “How often do you meet your family or family-in-law?”; and (3) “How often do you spend time on social leisure activities away from home?”, a category that included activities such as visits to catering facilities and hobbies in club-like environments. These interviewer-rated questions were answered on a scale ranging from 0 (daily), 1 (weekly), 2 (at least once a month), 3 (less than once a month), 4 (rarely never), to 5 (I don’t have friends/family/leisure activities). To constitute a formative measure for social involvement, the three frequency-related questions were selected from basic questions assessing social determinants (e.g., social contacts, socio-economic status, and working and living situation) that had been developed specifically for the UP’s study [30], and where each item reflected a specific, related but separate element of social involvement. The answers to these three questions were summarized on an index scale, with lower scores indicating more frequent interactions.

#### Personality traits

Neuroticism and extraversion were assessed using the NEO Five Factor Inventory *(*NEO-FFI-3) [8], whose neuroticism and extraversion scales both have acceptable internal consistency. Psychometric properties were also acceptable in a population of psychotic disorder patients [2]. For the neuroticism scale, Cronbach’s alpha was 0.842 and McDonald’s omega was 0.849; for the extraversion scale, the respective values were 0.743 and 0.725. Total mean scores were calculated from responses on a 5-point Likert-type scale ranging from 1 (strongly disagree) to 5 (strongly agree), with higher scores indicating higher levels of the specific trait. Missing values were not imputed.

### Analysis

IBM SPSS Statistics (Version 27) was used for all data analysis. Sociodemographic and patient characteristics were summarized, using means and standard deviations or percentages for categorial variables. Correlation analyses were performed using Pearson’s or Spearman’s correlation coefficients (*r or r*_*s*_*)* Assumptions for linear regression analyses were checked using scatter plots. To check whether the different social involvement questions could be put into one index-score with an equal weighting factor, sensitivity analyses were performed by repeating all regression analyses in different compositions (e.g., an index with question 1 and 2, versus 1, 2 and 3). Even though question 3 (time spent on social leisure activities) had a stronger correlation with PR than questions 1 and 2 (contact with friends and contact with family), the sensitivity analysis revealed very similar results.

To answer the research question, five regression models were conducted. Model 1 included the base model with the direct effect of social involvement on PR. In model 2, we added neuroticism but not extraversion to this base-model, and in model 3, we added extraversion but not neuroticism. To check for moderation effect of the personality traits, models 4 and 5 included the interaction effects of neuroticism and extraversion with social involvement. The incremental validity is indicated by the change in R-square when adding variables to the model.

## Results

### Descriptions of the study sample

At the time of data analysis, 320 participants had been included in the UP’S study. We included only those participants whose dependent and independent variables were complete (*N* = 284), leading to the exclusion of 36 participants with missing data. These 36 participants did not differ with respect to sample characteristics and baseline scores. The mean age, gender distribution, and living situation of the sample were representative of patients in treated in FACT [30]. At the time of assessment, participants had mild or no psychotic symptoms, and relatively good PR. The duration of care was relatively long, the mean duration being 14 years, representing a range from a few months to 37 years. For further details, see Table 1 below.

**Table 1:** Sample characteristics and baseline scores of participants in the study about the moderating effect of personality traits in the association between social involvement and personal recovery in psychosis

	Total sample (*N*=284) Mean/% (SD)	Valid N
Age	40.8 (12.23)	284
Gender (% male)	67	284
Employment status (% active in school/ work)	37	273
- Paid employment	14	
- Social Work provision Act and/or reintegration	7	
- School	3	
- Retired	1	
- Volunteer work	13	
- Incapacity	27	
- Unemployed	29	
Living situation (in %)		274
- Alone	51	
- With partner	13	
- With (partner and) children	5	
- With parents	13	
- In MHC institution	13	
- Other	2	
Diagnosis (in %)		284
- Schizophrenia	45	
- Schizoaffective disorder	9	
- Brief psychotic disorder	12	
- Substance-induced psychotic disorder	1	
- Schizotypical disorder	1	
- Psychosis NOS	25	
PANSS total (mean item-score, range 1-7)	2.0 (0.78)	272
- Positive symptoms	2.1 (1.05)	
- Negative symptoms	2.1 (1.05)	
- General symptoms	1.7 (0.85)	
ReQoL (total-score, range 0-40)	25.5 (7.47)	284
Social involvement (total score, range 0-15)*	5.52 (2.88)	284
- Interaction with friends (range 0-5)	1.78 (1.51)	
- Interaction with family	1.83 (1.39)	
- Time spent on leisure activities outdoor	1.91 (1.74)	
NEO-FFI Neuroticism (range 12-60)	37.0 (8.27)	284
NEO-FFI Extraversion (range 12-60)	37.0 (6.42)	284

*Higher scores indicate less frequent social interaction

### Regression analysis

Although associations with PR reached significance, the results of the linear regression analysis indicated that (1) only 6% of the variance in PR could be predicted by social involvement, (2) neuroticism was negatively associated with PR and 41% of the variance could be predicted when neuroticism was added to the model, and (3) extraversion was positively associated with PR and 16% of the variance of PR could be predicted when extraversion was added to the regression models. The effects of social involvement, neuroticism and extraversion on PR are shown in model 1–3 in Table 2 below.

To check for moderation, we added interaction-effects for neuroticism (b=−0.21, 95%CI= −0.55 to 0.13, *p* = 0.232) and extraversion (b = 0.01, 95%CI=−0.53 to 0.50, *p* = 0.956) to the association between social involvement and PR. However, this did not increase the explained variance in PR (see models 4 and 5 in Table 2, respectively).

**Table 2:** Results linear regression models of personal recovery (dependent variable) including social involvement, neuroticism and extraversion

	*b (95%CI)*	*SE B*	β	*p*	R^2^
Model 1					0.06
Constant	25.52 (24.67, 26.36)	0.43		<0.001	
Social involvement	-0.61 (-0.90, -0.31)	0.15	-0.24	<0.001	
Model 2					0.41
Constant	25.52 (24.85, 26.19)	0.34		<0.001	
Social involvement	-0.41 (-0.64, -0.13)	0.12	-0.16	<0.001	
Neuroticism	-6.55 (-7.53, -5.56)	0.50	-0.60	<0.001	
Model 3					0.16
Constant	25.51 (24.71, 26.31)	0.41		<0.001	
Social involvement	-0.41 (-0.69, -0.12)	0.16	-0.16	0.006	
Extraversion	4.69 (3.14, 6.24)	0.79	0.34	<0.001	
Model 4					0.42
Constant	25.58 (24.90, 26.25)	0.34		<0.001	
Social involvement	-0.41 (-0.65, -0.18)	0.16	-0.12	<0.001	
Neuroticism	-6,66 (-7.59, -5.62)	0.50	-0.61	<0.001	
Social involvement * Neuroticism	-0.21 (-0.55, 0.13)	0.17	-0.06	0.232	
Model 5					0.17
Constant	25.51 (24.68, 26.33)	0.42		<0.001	
Social involvement	-0.41 (-0.69, -0.12)	0.15	-0.16	0.002	
Extraversion	4.69 (3.14, 6.24)	0.79	0.34	<0.001	
Social involvement * Extraversion	0.01 (-0.53, 0.50)	0.26	-0.00	0.956	

Social involvement, neuroticism and extraversion were entered in the first three models, followed by the interaction effects between social involvement and neuroticism and extraversion respectively to check for moderation

Higher scores on social involvement indicate less frequent social interaction

## Discussion

The purpose of this exploratory cross-sectional study, was to gain insight into the association between social involvement and PR in people with psychosis, and also into the potential moderating effects of two personality traits, neuroticism and extraversion. Although social involvement was found to be significantly positively associated with PR, it could, as expected, explain only 6% of the variance in PR. Contrary to our hypotheses, however, neither of the personality traits moderated the association between social involvement and PR. As a result, the weak association between social involvement and PR observed in previous research could not be explained by a moderating effect of neuroticism and extraversion. Furthermore, after addition to the model, neuroticism appeared to have added a considerable proportion of unique variance in PR, while extraversion explained only a modest amount of unique variance.

Despite the growing number of social interventions aiming to increase PR through processes such as enhancing connectedness, and to tackle themes such as social isolation, and perceived social stigma, relatively few studies have explicitly investigated the association between social factors and PR. The relatively weak associations found to date [18], were confirmed by our finding that only a small proportion of the variance in PR was explained by social involvement, defined as the quantity of social interactions in our study. However, PR may in fact depend more on *the quality* of these interactions than the quantity. Not only has the importance of the quality of interaction and social support been stressed in studies of patient accounts of PR in psychosis [37], there is also some evidence that, in psychosis, the extent of *self-reported* interpersonal interaction and prosocial activities is associated with PR [33]. Our reason for using an interviewer-rated quantitative measure, was based on our desire to avoid conceptual overlap with patient-rated PR (e.g., connectedness). We therefore suggest that future studies assess the quantity and quality of social involvement simultaneously.

Our tentative finding that neuroticism plays a strong negative role in PR, is in line with previous research findings that high levels of neuroticism in people with psychosis were a strong predictor of quality of life [13], and that these effects were stronger in patients with psychosis than in controls [24]. Neuroticism indicates a susceptibility to negative affect and psychological distress. Affective symptoms consistently have a stronger association with PR than psychotic symptoms do [18, 32]; and, in patients with psychosis, negative affect has a stronger negative association with subjective quality of life than in controls [17]. In line with these studies, and more recent findings in individuals at risk for psychosis [6], our results suggest that PR in people with psychosis may be enhanced by targeting negative emotions and underlying stress factors. Together with our suggestion that PR may depend more on the quality of social interactions, it is possible that PR will be promoted by interventions such as social-skills training, which not only has a direct impact on the quantity of social interactions, but also on the quality, and an indirect impact on negative symptomatology [28].

We hypothesized that the small association between social involvement and PR would be facilitated by extraversion, which indicates higher degrees of sociability, positive emotionality and general activity. With regard to PR, we expected that more extraverted patients would tend to place a stronger emphasis on social involvement than patients with lower extraversion scores would. However, although the impact of extraversion on social functioning is well-established in the general population, this association is more ambiguous in people with psychosis; while some studies find no association [11, 19, 24], other studies indicate a stronger role for extraversion in social functioning [6]. One explanation for this ambiguity might be the fact that social involvement is a broad construct measured in different ways; in the study of Lysaker and Davis [19] an interviewer rated instrument was used, while Boyette et al [6] used a self-reported questionnaire. Moreover, it has been speculated that the positive effects of extraversion on sociability are nullified by high levels of public and perceived stigma in people with psychosis [19]. It is also the case that, like quality of life, PR is a highly subjective construct, which may represent a strong network of close relationships for one person, whereas another person may forego such relationships in favor of personally meaningful activities or accomplishments, such as professional advancement or creative pursuits [12, 16]. Future studies should take account not only of such diversities in social involvement and PR, but also of the potential role of public stigma in psychosis.

### Limitations

Although the weak association we observed between social involvement and PR is in line with previous research, social involvement, PR, and personality traits are all complex constructs. Our results should therefore be viewed both with caution and with reference to the following potential methodological issues.

Firstly, the fact that we did not assess social involvement with a specific and validated questionnaire is an important limitation in our study. However, low associations were also found between social involvement and PR by several previous studies that *did* use validated questionnaires for this purpose [18]. Nevertheless, other aspects of social functioning such as workplace, vocational engagement and daily routines like shopping for groceries have been neglected in this study and should be included in future research.

The fact that we found no associations between variables may have been due to the characteristics of this psychosis sample, which was characterized by low levels of current psychotic symptoms, and relatively high levels of PR and social interaction. Perhaps only the more symptomatically recovered patients agreed to participate in the study, however, symptom severity in our sample was very similar to that of a study with a resembling population [35]. Although scores on neuroticism and extraversion both showed low variance in our sample, the mean scores and dispersion of the two traits were equally high, and both resembled those of similar samples [11, 31].

Another limitation is that we did not look at interactions between traits in personality profiles, but included neuroticism and extraversion as separate traits. It is conceivable that the combination of traits (e.g., low extraversion in combination with high neuroticism) had a stronger impact than the effect of the two traits separately. However, this was not confirmed in the study by Ridgewell et al. [24], which, both separately and in combination, examined the effects of neuroticism and extraversion on quality of life and overall functioning in people with psychosis, and found no difference between the two approaches. These findings, together with the low variance in neuroticism and extraversion scores in our sample, made us decide not to include extra analyses with combination of traits. However, future research should take this into account.

Finally, regarding the use of ReQoL-10 as a PR-questionnaire, the low association with social involvement may have been due to the possibility that the ReQoL-10 places less emphasis on the CHIME process “Connectedness,” as only 1 out of 10 items (“I feel lonely”) reflected connectedness. An earlier meta-analysis, which examined whether the type of PR-questionnaire could explain any heterogeneity between studies regarding the associations between social factors and PR, found that such an influence was marginal [18].

Our results give rise to four main points of interest for future research. The first stems from the fact that our moderation analysis did not include correction for factors such as negative symptoms and negative affect. Since neuroticism indicates susceptibility to negative affect and psychological distress, we assumed that it might be a reflection of these factors. Future studies should address this.

Second, this was a cross-sectional study. However, clinically relevant answers about causality may be provided by longitudinal research that examines the effect of personality traits—especially neuroticism—on the association between social involvement and PR over time in psychotic disorders. Although one study found that the association between neuroticism and subjective well-being remained stable over time [31], more longitudinal research may answer questions such as whether the courses of personal and symptomatic recovery are influenced by a high degree of neuroticism, or whether personality traits are affected by poor recovery. Such research may reveal why trajectories of recovery differ between people, and thus lead to the development of more personalized treatment plans.

Third, although the duration of care in our psychosis sample was relatively long, with a wide range of durations, its distribution did not allow for separate analyses that would make it possible to compare an early-episode group of patients (e.g., the first five years after a first episode of psychosis) with a more chronic group. Perhaps the moderating effect of personality traits increases over time in treatment. Differentiating between an early episode and a longer duration of care group would be all the more interesting, as personality traits generally become stable when individuals reach their early thirties [9].

Finally, questions about the impacts of stigma and rejection in the association between social involvement and PR are raised not only by our finding that extraversion added only a moderate amount of unique variance to the model of PR and social involvement, but also by the ambiguity of findings on the association between extraversion and social functioning in psychosis in the earlier literature [19]. Future research in this area might reveal that facilitating PR in psychosis is partly a question of addressing the image of psychosis that prevails in society [38]*.*

### Conclusion

This study showed that levels of social involvement are only marginally associated with PR, and that this lack of a strong association cannot be explained by a moderating effect of neuroticism and extraversion. Our finding that the explained variance of PR was increased particularly by the addition of neuroticism to the model of social involvement and PR, is in line with a large body of literature on the effects of neuroticism on QoL, and illustrates the importance, when treating people with psychosis, of attending to their negative affect and underlying distress. Attending to negative affect and underlying stressors actually constitutes the main target point of several interventions, such as cognitive behavior therapy for psychosis. As our results are based on the quantity of social interactions, they do not undermine the potential importance of the quality of social interaction that patients emphasize in their accounts of PR from psychosis, or of the support they obtain from it.

## Data Availability

Data is available upon reasonable request from the corresponding author.
